# Deep Eutectic Solvent-Based Ultrasound-Assisted Extraction of Flavonoids from *Houttuynia cordata*

**DOI:** 10.3390/foods14040558

**Published:** 2025-02-07

**Authors:** Xinxin Wu, Ling Yan, Jingda Li, Zhijian Tan

**Affiliations:** 1 College of Life Sciences, Yangtze University, Jingzhou 434025, China; wuxinxin20242024@163.com; 2 Institute of Bast Fiber Crops and Center of Southern Economic Crops, Chinese Academy of Agricultural Sciences, Changsha 410205, China; yanling7711@126.com; 3 College of Life and Environmental Science, Hunan University of Arts and Science, Changde 415000, China

**Keywords:** flavonoids, deep eutectic solvents, response surface optimization, biological activity

## Abstract

Recently, deep eutectic solvents (DESs) have attracted much attention in the extraction and separation field because of their green characteristics, and they are widely used to extract various bioactive substances from plants. In this study, ten choline chloride (ChCl)-based mixtures were selected for extracting flavonoids from Houttuynia cordata. Then, the optimal conditions for the DES-based extraction of *Houttuynia cordata* flavonoids (HCFs) were determined through one-way experiments and response surface optimization (RSM). The optimal conditions were a 60 min extraction time, an extraction temperature of 40 °C, a water content of 30%, a solid−liquid ratio of 1:22 g·mL^−1^, a molar ratio of 1:2, and an ultrasound power of 420 W. The antioxidant capacities of HCFs in terms of ABTS radical, DPPH radical, and hydroxyl radical scavenging capacity and nitrite inhibition capacity were determined. DESs can be efficiently recycled after extraction. This study developed an effective and environmentally friendly method for HCF extraction, thereby also supporting the development and utilization of natural products.

## 1. Introduction

Flavonoids are among the most important compounds found in plants, and they have a range of bioactivities, such as antioxidant activity, anticancer activity, tumor-inhibiting capacity, and cardiovascular protection activity [1,2]. However, flavonoids in natural foods may be present in complex matrices, and they need to be released to increase their bioavailability and make them more readily absorbed and utilized by the body. In addition, obtaining flavonoids directly from plants is a relatively more environmentally sustainable method than chemical synthesis [3,4]. *Houttuynia cordata,* a member of the *Saururaceae* family, is a well-known medicinal and edible plant with homeopathic uses. It is widely distributed in China south of the Yangtze River basin, India, Japan, Korea, and other regions of Asia [5,6]. It smells pungent and cold and has the pharmacological actions of heat detoxification, diuretic swelling, and stomachic effects. These actions are attributed to its main ingredients of organic acids, volatile oils, and flavonoids, among which flavonoids are rich in terms of both type and content. Studies have shown that *Houttuynia cordata* contains four major flavonoids, namely, rutin, quercetin, isoquercitrin, and hyperin [7,8]. Due to their pharmacological diuretic, antibacterial, anticancer, antioxidant, and antimutagenic effects, *Houttuynia cordata* flavonoids (HCFs) have been used to treat a variety of diseases [9,10,11].

Accelerated solvent extraction (ASE), supercritical fluid extraction (SFE), microwave-assisted extraction (MAE), enzyme-assisted extraction (EAE), and ultrasound-aided extraction (UAE) are frequently employed techniques used for extracting flavonoids [12]. Ultrasound extraction is an effective, secure, and economical extraction technique that can produce a cavitation effect, thus effectively destroying plant cell walls so that bioactive substances are readily released into the extraction solvent [13]. Currently, the solvents used in conjunction with ultrasound assistance are usually organic solvents, for example, ethanol, methanol, and acetone. Nonetheless, organic solvents frequently have numerous downsides, such as their possible volatility, flammability, biotoxicity, and even residues in the extract, restricting the sustainability of the extraction process [14]. Therefore, there is an urgent need to find an effective and green alternative solvent for the extraction of HCFs.

Deep eutectic solvents (DESs) are mixtures with low melting points formed through hydrogen bonding interactions between a hydrogen bond donor (HBD) and a hydrogen bond acceptor (HBA). They have the advantages of excellent biodegradability, lower costs, simple preparation, and negligible biotoxicity [15,16]. Thus, DESs are extensively utilized to extract active ingredients from plants, representing a new generation of environmentally benign and green solvents with the potential to replace organic solvents [17]. Sun et al. used DESs (choline chloride/ethylene glycol, 1:2) as alternate solvents for the extraction of flavonoids from hawthorn (Crataegus pinnatifida) with higher yields [18]. Lin et al. established a method based on DESs (L-proline/glycerol, 2:5) for the extraction of polyphenols from *Chenopodium quinoa* Willd [19]. Wang et al. extracted total flavonoids from *Perilla frutescens* (L.) Britt. leaves using DESs (glucose/glycerol, 1:4.3), where a significantly higher extraction yield was obtained compared to conventional extraction using ethanol; moreover, the DES extract exhibited better biological activity [20].

This study was carried out to screen for the best DESs for HCF extraction out of ten DESs. Response surface optimization (RSM) experiments were utilized to optimize the critical conditions influencing HCF extraction. The in vitro antioxidant capability and nitrite inhibition activity were determined for HCFs.

## 2. Materials and Methods

### 2.1. Reagents and Materials

*Houttuynia cordata* was collected from Changde, Hunan Province, China. Rutin (HPLC ≥ 98%) and sodium citrate buffer (0.1 mol·L^−1^, pH 3.0) were obtained from Shanghai Yuanye Biotechnology Co., Ltd. (Shanghai, China). Choline chloride (ChCl), urea, ethylene glycol, 1,3-propanediol, glycerol, 1,4-butanediol, and oxalic acid were purchased from Adamas Beta Reagent Co., Ltd. (Shanghai, China). 1,1-Diphenyl-2-picrylhydrazyl (DPPH), 2,2′-Azino-bis (3-ethylbenzothiazoline-6-sulfonic acid) (ABTS), lactic acid (La), malonic acid, malic acid, citric acid, and ethanol (ET) were obtained from Shanghai Aladdin Reagent Co., Ltd. (Shanghai, China).

### 2.2. Sample Preparation

Fresh leaves of *Houttuynia cordata* were thoroughly washed to remove any sediment. The cleaned leaves were then dried in an oven at 50 °C until a stabilized weight was achieved. For future use, the dried leaves were ground and passed through a 50-mesh sieve and then sealed in a container and stored away from light to prevent degradation.

### 2.3. Preparation of DESs

HBAs and HBDs were mixed at a 1:1 molar ratio, and the mixture was stirred at 80 °C until forming a homogeneous clear liquid [21]. The prepared mixture was vacuum-dried to an anhydrous state, after which water was added at a mass ratio of 20% [22]. The density of the DESs was determined by the density bottle method (ρ = m/V), and the viscosity of the DESs was determined by rheometry (MCR 92). The DESs were analyzed using Fourier transform–infrared spectroscopy (FT-IR) (Thermo Nicolet iS5) and nuclear magnetic resonance spectroscopy (NMR) (Bruker 400 M) [23]. 

### 2.4. Extraction of HCFs

A volume of 6.0 mL of DESs was added to Houttuynia cordata leaf powder (0.2 g) in a 50 mL test tube. Sn ultrasound instrument (KQ700DE, Kunshan Ultrasonic Instrument Co., Ltd., Kunshan, China) was used for 30 min at 350 W and 40 °C to extract the flavonoids. The flavonoid solution was then collected by centrifugation. The total flavonoid content was evaluated using a UV–visible spectrophotometer (UV3000PC, Shanghai Mepda Instrument Co., Ltd., Shanghai, China) with the sodium nitrite-aluminum nitrate colorimetric method [24]. The extraction yield was determined using Equation (1). Figure S1 shows the standard curve for rutin measurement.

(1) $$\mathrm{Extraction}\;\mathrm{yield}\;\left(\mathrm{mg}\cdot g^{-1}\right)=\frac{C\times V}{M}$$

where *C* is the total flavonoid concentration in the *Houttuynia cordata* leaf powder (mg·mL^−1^), *V* is the extracted liquid volume (mL), and *M* is the quantity of *Houttuynia cordata* leaf powder (g).

### 2.5. Single-Factor Experiment

This study examined how the water content in DESs (10–50%), the solid–liquid ratio (1:10–1:50 g‧mL^−1^), the ultrasound power (350–630 W), the extraction time (30–90 min), the molar ratio of HBA and HBD (1:1–1:5) (detailed information is provided in Table 1, ^13^C NMR data are shown in Figure S3), and the temperature (30–70 °C) affect the extraction yield. Table 1 provides details of the DESs with different molar ratios, and Figure S3 shows the ^13^C NMR data of the DESs with different molar ratios.

### 2.6. RSM Experiments

Based on the single-factor experiment results, the interactions between three major variables were examined using a Box–Behnken design (BBD). To maximize the HCF extraction, a three-factor, three-level BBD experiment was carried out. In light of the experimental results, 17 sets of tests were designed to analyze the interactions, with the solid–liquid ratio (A), molar ratio (B), and ultrasonic power (C) serving as the independent variables and the HCF extraction yield as the response value. The experimental program is presented in Table S1. The second-order multinomial model of the HCF extraction yield is shown below (Equation (2)):

(2) $$Y=\beta_{0}+\sum_{i=1}^{3}\beta_{i}X_{i}+\sum_{i=1}^{3}\beta_{ii}X_{i}^{2}+\sum_{i=1}^{2}\sum_{j=i+1}^{3}\beta_{ij}X_{i}X_{j}$$

where Y is the response variable; $\beta_{0}$ is a constant; β, $\beta_{ii}$, and $\beta_{ij}$ are the model’s main, secondary, and interaction variables, respectively; and $X_{i}$ and $X_{j}$ are independent variables.

### 2.7. DES Recycling Experiment

HCFs were extracted using DES, and the extract was then adsorbed and desorbed (ethanol) by macroporous resin (AB-8) to produce an HCF–ethanol solution and a DES recovery solution, respectively [25]. The HCFs were then produced by freeze-drying the HCF–ethanol solution. The recovered DES was used in the subsequent HCF extraction, which was carried out five times.

### 2.8. Antioxidant Capability

#### 2.8.1. DPPH Scavenging Capability

A DPPH solution with a volume of 2.0 mL was mixed with 1.0 mL of various concentrations of Houttuynia cordata leaf extract (0.2–1.0 mg·mL^−1^) [26]. The absorbance at 517 nm was measured after 30 min of remaining in the dark (SpectraMax Mini, Meigu Molecular Instruments Co., Ltd., Shanghai, China). The scavenging rate of DPPH was calculated according to Equation (3).

(3) $$\mathrm{DPPH}\;\mathrm{radical}\;\mathrm{scavenging}\;\mathrm{activity}\;(\%)=[1-(A_{i}-A_{j})/A_{0}]\times 100\%$$

where $A_{i}$ represents the sample’s absorbance following the reaction reaching equilibrium, $A_{j}$ represents the sample itself (sample + 95% ethanol), and $A_{0}$ represents the absorbance of the DPPH radical in the absence of an additional sample (95% ethanol + DPPH–ethanol solution).

#### 2.8.2. ABTS Scavenging Capability

A 1.0 mL sample with varying concentrations was obtained and thoroughly combined with 2.0 mL of ABTS working solution (0.2–1.0 mg·mL^−1^) [27]. The absorbance at 734 nm was measured after 6 min of remaining in the dark. Equation (4) was used to calculate the ABTS scavenging rate.

(4) $$\mathrm{ABTS}\;\mathrm{radical}\;\mathrm{scavenging}\;\mathrm{activity}\left(\%\right)=\left[1-A_{i}^{\prime }-A_{j}^{\prime })/A_{0}^{\prime }\right]\times 100\%$$

where $A_{i}^{\prime }$ represents the sample’s absorbance following the reaction reaching equilibrium, $A_{j}^{\prime }$ represents the sample itself (sample + 95% ethanol), and $A_{0}^{\prime }$ is the ABTS radical absorbance without sample addition (95% ethanol + ABTS–ethanol solution).

#### 2.8.3. Hydroxyl Scavenging Capability

Ferrous sulfate (1.0 mL at 9.0 mmol‧L^−1^) and 1.0 mL of salicylic acid (9.0 mmol‧L^−1^) were added to test tubes containing 1.0 mL of samples at different concentrations (0.2–1.0 mg·mL^−1^) and mixed thoroughly. After the addition of one milliliter of hydrogen peroxide solution, the solution was held in a water bath at 37 °C for 30 min, and the absorbance was measured at 510 nm [27]. Using Equation (5), the hydroxyl scavenging rate was determined.

(5) $$\mathrm{Hydroxyl}\;\mathrm{radical}\;\mathrm{scavenging}\;\mathrm{activity}\left(\%\right)=\left[1-(A_{i}^{\prime \prime }-A_{j}^{\prime \prime })/A_{0}^{\prime \prime }\right]\times 100\%$$

where $A_{i}^{\prime \prime }$ represents the sample’s absorbance, $A_{0}^{\prime \prime }$ represents the absorbance measured using distilled water in place of the sample, and $A_{j}^{\prime \prime }$ represents the absorbance measured using distilled water in place of hydrogen peroxide.

### 2.9. Nitrite Scavenging Capacity

Samples at a volume of 1.0 mL with different concentrations were taken and mixed thoroughly with 1.0 mL of NaNO_2_ (1.0 mM), 8.0 mL of sodium citrate solution (pH 3.0), and 2.0 mL of 0.4% p-aminobenzene sulfonic acid solution for 5 min (0.02–0.1 mg·mL^−1^). A 0.2% solution of naphthalene ethylenediamine hydrochloride was added and allowed to stand for 15 min, after which the absorbance was measured at 540 nm [28]. The nitrite scavenging capacity rate was calculated using Equation (6).

(6) $$\mathrm{Nitrite}\;\mathrm{scavenging}\;\mathrm{activity}\left(\%\right)=\left[1-(A_{1}-A_{2})/A_{3}\right]\times 100\%$$

where $A_{1}$ represents the sample’s absorbance following the reaction reaching equilibrium, $A_{2}$ indicates the absorbance of the sample, and $A_{3}$ represents the absorbance measured using distilled water instead of the sample.

### 2.10. Statistical Analysis

The results are presented as averages, with each test performed in triplicate. Data analysis was conducted using IBM SPSS Statistics 26. ANOVA was applied to examine the results, and significant differences (*p* < 0.05) are indicated by different letters

## 3. Results and Discussion

### 3.1. Characterization of DESs

**Figure 1:**
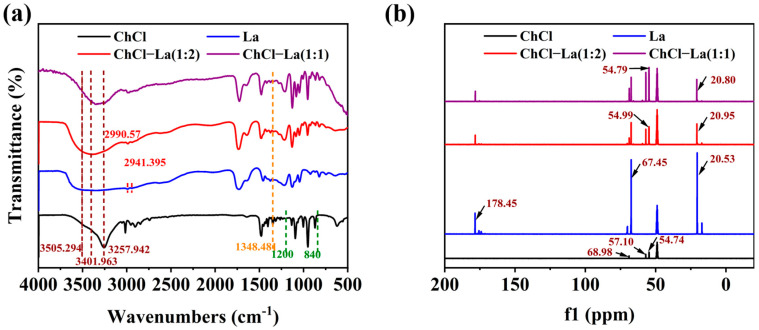
The FT-IR spectra of ChCl, La, and ChCl−La (**a**) and the ^13^C NMR spectra of ChCl, La, and ChCl−La (**b**).

Firstly, the structures of the DESs were characterized. The chemical structure of the DESs were analyzed by FT-IR. As shown in Figure 1a, which is the FT-IR plot of ChCl−La, the peak at 1200–840 cm^−1^ corresponded to the N-H and C-N bonds, and the vibrational band at 1348.481 cm^−1^ disappeared after the synthesis of the DESs, thus proving the successful synthesis of the DESs. Furthermore, in the FT-IR diagram of La, the vibrational bands at a wave number of 3505.294 cm^−1^ corresponded to the bending vibration of -OH, whereas the vibrational bands at wave numbers of 2990.57 cm^−1^ and 2941.395 cm^−1^ corresponded to the stretching vibration of CH_3_. After the synthesis of the DESs, the -OH vibrational band of lactic acid moved to the left (3401.963 cm^−1^), which may have been brought about by choline chloride and lactic acid forming hydrogen bonds. This shift indicates that the synthesis of the DESs was successful [29,30,31]. The ^13^C NMR (deuterium methanol) data of DES also proved its successful synthesis. As shown in Figure 1a, the peaks at 54.74, 57.10, and 68.98 ppm in ChCl represent -CH_3_, -CH_2_OH, and -NH_2_CH_2_, respectively [32]. The peaks at 20.53, 67.45, and 178.45 ppm in La represent -CH_3_, -COOH, and -CH(CH_3_), respectively [33]. After synthesizing ChCl−La (1:1–2), δ 54.79 (m,17C) and 54.99 (m,17C) represent -CH_3_ in the ChCl, and δ 20.80 (s,5C) and 20.95 (s,9C) represent -CH_3_ in La, respectively, and this result proves that the ratios of ChCl and La were 1:1 and 1:2. FT-IR and ^13^C NMR maps of the other DESs are shown in Figure S2.

### 3.2. Selection of the Optimal DESs

In this work, ChCl was selected as the HBA, and ten extraction solvents were prepared. Table 2 provides comprehensive information regarding the DESs. Extraction using 60% ET was used as a control. Table 2 shows that the ChCl−La had the maximum extraction yield, and the majority of the DESs had a better extraction efficiency than ethanol or a comparable extraction efficiency. It has been reported that when DES is used as an extraction solvent, not only do its physical properties (viscosity, solubility, polarity, and diffusivity) but also the interaction forces (hydrogen bonding, hydrophilicity, van der Waals’ forces, and electrostatic interactions) between the DES and the target substances affect the extraction efficiency [34,35]. In other words, a combination of several of these parameters affects the extraction efficiency of DES. ChCl−La was the most effective DES in this trial.

### 3.3. Single-Factor Experiment Analysis

**Figure 2:**
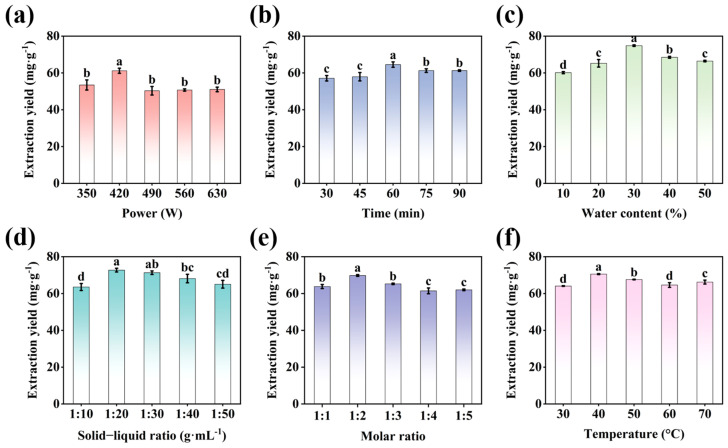
The impact of extraction parameters on the extraction yield of HCFs. (**a**) Ultrasonic power, (**b**) extraction time, (**c**) water content of DESs, (**d**) solid−liquid ratio, (**e**) molar ratio, and (**f**) extraction temperature. Values sharing common superscripts were not statistically different.

Figure 2a shows the influence of varying the ultrasonic power on the extraction yield. Ultrasound’s cavitation, mechanical, and thermal effects can break cell walls and increase molecular mobility, allowing for the fast release and dissolution of flavonoids [18]. The mechanical action of ultrasound increases with a larger power. When the ultrasound power was raised by 350 to 630 W, the first increase was followed by a fall, which was mostly because too much ultrasonic power generated too much heat, killing some heat-sensitive flavonoids and resulting in a loss of extraction yield [36]. Therefore, the optimal ultrasound power was 420 W.

As the extraction duration increased, the extraction yield first exhibited an increasing trend before declining, as illustrated in Figure 2b. This was mainly because the extraction time was too short (30–45 min), the contact between the DESs and *Houttuynia cordata* powder was not sufficient, and many active compounds were not dissolved. The lower extraction rate with the longer extraction time could be attributed to the dissolution of an enormous amount of impurities, which limited the dissolution of the HCFs [13,37]. Therefore, the optimal extraction time was 60 min.

The system’s water content affected the viscosity of the DESs and, thus, the extraction efficiency [38]. As water content of the DESs increased, the extraction yield first exhibited a rising trend before declining, as illustrated in Figure 2c. This may be because the core action of DESs involves hydrogen bonding. As the proportion of water increased, the hydrogen bonding within the DESs was enhanced, leading to an increased depolymerization of flavonoids. However, excessive addition of water (> 30%) disrupted the hydrogen bond interactions, ultimately decreasing the dissolution of the target substances [39]. Therefore, an optimal water content of 30% was chosen.

The extraction yield initially rose and subsequently dropped as the amount of liquid increased, as shown in Figure 2d. The low liquid ratio caused the agglomeration of the *Houttuynia cordata* leaf powder, which was thus not sufficiently mixed with the DESs, resulting in insufficient flavonoid dissolution. A high proportion of liquid resulted in a lower extraction yield, most likely because the excess solvent absorbed heat and made the mixture more viscous, which is not ideal for extracting flavonoids [40]. Therefore, the optimum solid–liquid ratio was 1:20 g·mL^−1^.

The molar ratio of HBA to HBD can be changed to modify the viscosity, surface tension, and hydrogen bonding of DESs [41]. The extraction yield at various molar ratios rose and subsequently fell, as shown in Figure 2e. This could be because different molar ratios of DESs have different characteristics, like the stability and strength of the hydrogen bonding interactions [42]. Therefore, the optimal molar ratio was 1:2.

As the temperature rose, the flavonoid extraction yield first increased and subsequently decreased, as shown in Figure 2f [36]. When the temperature was too low, the molecular movement was retarded, resulting in a decrease in dissolution, diffusion, and osmosis, which was not facilitated by the dissolution of the flavonoids. Excessive temperatures can disturb the structure of a protein or substance, leading to the degradation of heat-sensitive chemicals in it, causing the extraction yield to decline [43,44]. Therefore, the optimal extraction temperature was 40 °C.

### 3.4. RSM Experiments Analysis

The major parameters of the solid–liquid ratio (A), DES molar ratio (B), and extraction power (C) were optimized as independent variables, and the total flavonoid yield was determined as the response value at a fixed extraction temperature of 40 °C, water content of 30%, and ultrasound time of 60 min. A quadratic polynomial was fitted to the data in Table S2, and the second-order regression equation for the fitted model is given by Equation (7).

(7) $$\begin{array}{ll} Y= & -147.48659+3.65928\;A+25.43306\;B+0.771850\;C+0.071010\;AB-0.000013\;AC\\ & -0.002520\;BC-0.088814\;A^{2}-6.40471\;B^{2}-0.000901\;C^{2} \end{array}$$

According to Table S3, the model was considered significant if the “model *p*-value” was less than 0.0001. The “*p*-value of the misfit term” was 0.1485, which suggests that the “misfit term” was not significant concerning the pure error. It can be shown that A, A^2^, B^2^, and C^2^ had a highly significant effect on the extraction yield (*p*-value < 0.01). In addition, the “R_Pred_^2^” value of 0.9519 was reasonably consistent with the “R_adj_^2^” value of 0.9906. Thus, the model can be used to investigate and predict HCF extraction. The “*p*-value” shows that the influence of each element on the HCF extraction yield was as follows: solid–liquid ratio > ultrasonic power > molar ratio, in descending order.

**Figure 3:**
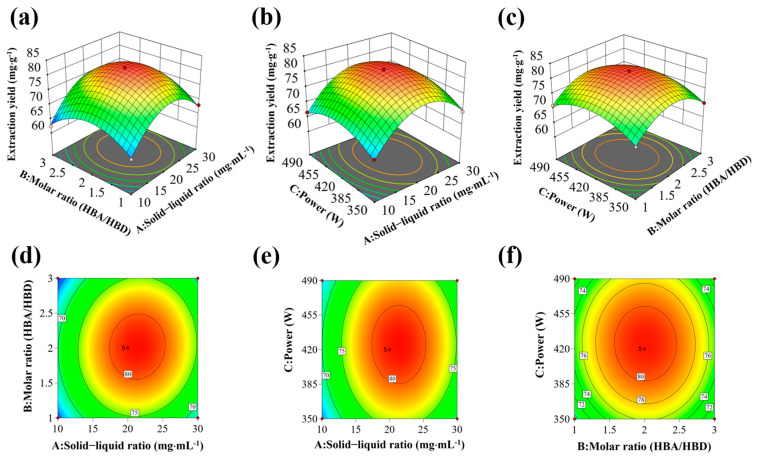
RSM plots of extracted HCFs. (**a**,**d**) Solid−liquid ratio and molar ratio, (**b**,**e**) solid−liquid ratio and sonication power, (**c**,**f**) molar ratio and sonication power. The faster the color change, the more significant the impact on the results.

Figure 3 displays the 3D response surface plots of the flavonoid extraction factors. Based on a quadratic model, the optimal parameters were a solid–liquid ratio of 1:21.38 g·mL^−1^, a molar ratio of 1:2.02, and a power of 425.22 W; the predicted extraction yield was 81.42 mg·g^−1^. Three replicate experiments were conducted at a solid–liquid ratio of 1:22 g·mL^−1^, a molar ratio of 1:2, and a power of 420 W to confirm the model’s dependability, which resulted in an extraction yield of 79.86 mg·g^−1^ of HCFs. The reliability of the ideal parameters found in this experiment is demonstrated by the experimental values’ proximity to the expected values. Under these circumstances, the flavonoids that were isolated from *Houttuynia cordata* leaves were significantly more effective than those that were extracted by Li et al. (32.4 mg·g^−1^) [45]. The cyclability of the DESs was also evaluated under ideal circumstances, and Figure S4 demonstrates that after five cycles, the extraction rate of HCFs only dropped by 11.01 mg·g^−1^, suggesting that the DESs have good cycling stability.

### 3.5. SEM Analysis

**Figure 4:**
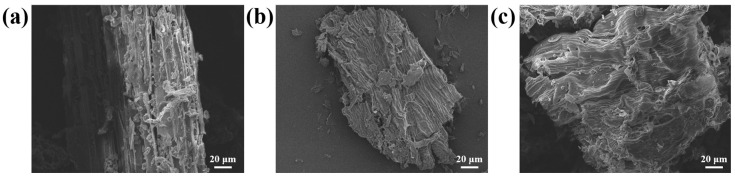
SEM images of *Houttuynia cordata* leaf powder: (**a**) untreated, (**b**) DES-treated, and (**c**) 60% ET-treated post-*Houttuynia cordata* leaf powder; 500× magnification.

Flavonoids and other bioactive components are usually distributed in different cells of plants, and the main factor hindering the diffusion of molecules between or within plant cells is the cell wall, so the selection of suitable extraction methods to break the cell wall to obtain target compounds is a key factor in improving the extraction performance [46,47]. The current study used an SEM (SEISS Sigma 300, Carl Zeiss Shanghai Management Co., Ltd., Shanghai, China) at a magnification of 500 to observe modifications to the morphology and architecture of *Houttuynia cordata* powder before and following extraction with DESs and organic solvents. As illustrated in Figure 4a–c, the cell walls of unextracted cells of the plants were in a whole-block configuration; however, following extraction by various techniques, the surfaces of the plant cells were concave and seemed fibrous due to the degradation of the walls of the cells by the extraction solvents. In particular, the cell surfaces of the treated *Houttuynia cordata* powder were severely damaged, probably because the DESs were capable of dissolving the lignin within the plant walls of the cells. A large amount of flavonoids flowed out of the cells during the HCF extraction process, which increased the extraction rate of HCFs [48].

### 3.6. Antioxidant Capacity and Inhibition of Nitrite Capacity of HCFs

**Figure 5:**
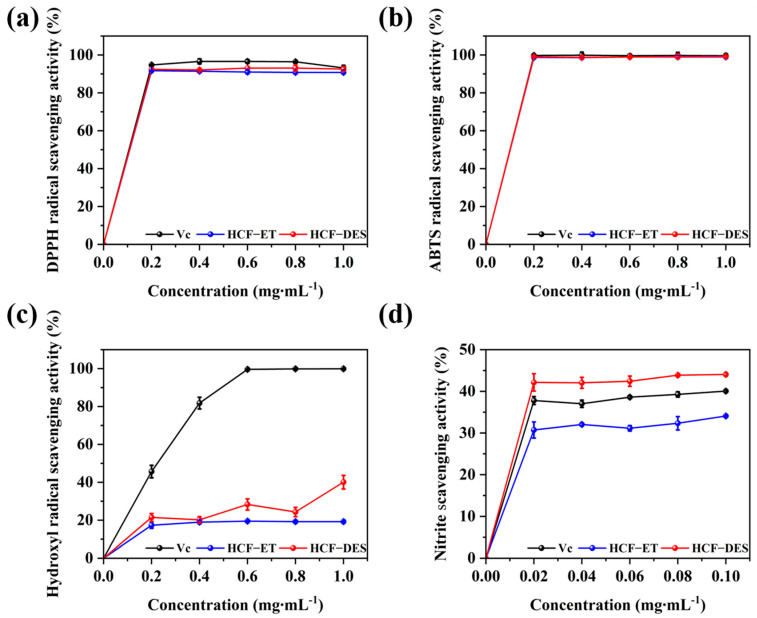
The scavenging capacity of HCF extract. (**a**) DPPH radical scavenging activity, (**b**) ABTS radical scavenging activity, (**c**) hydroxyl radical scavenging activity, (**d**) nitrite scavenging activity.

The most commonly used technique for evaluating the in vitro antioxidant capacity of natural extracts is an assay that measures the free radical scavenging activity. In vitro assessments of antioxidant capacity are essentially based on the antioxidants’ efficacy in terms of scavenging free radicals and lowering pro-oxidant capacity [49]. DPPH radical scavenging capacity, ABTS radical scavenging capacity, and hydroxyl radical scavenging capacity were used in this investigation to assess the antioxidant capacity of the HCFs. The capacity of the HCFs to scavenge DPPH, ABTS, and hydroxyl radicals improved with increasing the concentration, as illustrated in Figure 5a–c. When the sample concentration was 1.0 mg·mL^−1^, the greatest scavenging rate was noted, where the scavenging rates of DPPH, ABTS, and hydroxyl radicals by the HCFs extracted with DESs were 93.10%, 99.18%, and 40.60%, respectively, which were higher than that of 60% ET-extracted HCFs on DPPH (91.68%), ABTS (98.95%), and hydroxyl radicals (19.48%). The reason for these results could be that the DESs extracted more plant bioactive compounds, which resulted in higher antioxidant activity than organic solvent extraction [50].

As shown in Figure 5d, the DES-extracted HCFs had a better nitrite scavenging ability than that of the alcohol-extracted HCFs, which was 9.98% higher. It was discovered that the flavonoid and polyphenol concentrations had a positive impact on the antioxidant and nitrite scavenging capabilities [51]. Natural fruits, vegetables, and plants are abundant in phenols and flavonoids, two types of antioxidants that boost the body’s activity of catalase and SOD (superoxide dismutase) and stop nitrosamines and nitrites from being produced [52].

## 4. Challenges and Prospects

The DESs and optimization methods employed in this investigation demonstrated outstanding extraction efficiency, as indicated in Table S4. Ultrasound-assisted DES extraction of flavonoids is efficient and mild, and it can efficiently extract heat-sensitive flavonoids; nevertheless, the viscosity of DESs is substantially higher than that of commonly employed standard solvents, complicating subsequent solid−liquid separation [53]. Furthermore, it is difficult to separate target chemicals from DESs because of their low vapor pressure. The most widely used technique that has been documented is macroporous resin chromatography, which is easy to use and inexpensive, although it is challenging to accomplish effective separation. Finding a quick and effective separation technique is therefore essential to the advancement and application of DESs [53,54].

## 5. Conclusions

This study successfully extracted HCFs using a green and effective DES-based extraction technique. The extraction conditions were optimized using RSM, and the optimal parameters were as follows: a solid−liquid ratio of 1:22 g·mL^−1^, a molar ratio of 1:2, an extraction period of 60 min, an extraction temperature of 40 °C, a water content of 30%, and an ultrasound power of 420 W. Under optimal circumstances, the highest extraction yield reached 79.86 mg·g^−1^. Furthermore, in comparison to traditional alcohol extract, the HCFs derived from DES extract demonstrated greater DPPH scavenging capacity, ABTS scavenging capacity, hydroxyl scavenging capacity, and suppression of nitrite capacity. Moreover, the HCFs extracted by DESs were able to maintain good bioactivity, demonstrating potential for further applications in the food and pharmaceutical fields.

## Figures and Tables

**Table 1:** Detailed information regarding the DESs with different molar ratios.

No.	Solvent Composition		Molar Ratio	Water Content	Viscosity (mPa·s)	Density (g·mL^−1^)
	HBAs	HBDs				
1	Choline chloride	Lactic acid	1:1	20%	29.01 ± 0.11 ^a^	1.18
2			1:2	20%	20.42 ± 0.11 ^b^	1.17
3			1:3	20%	14.85 ± 0.15 ^c^	1.18
4			1:4	20%	14.34 ± 0.13 ^d^	1.19
5			1:5	20%	13.43 ± 0.15 ^e^	1.20

Note: viscosity measurement was repeated three times and is expressed as mean ± standard error; different letters indicate significant differences (*p* < 0.05).

**Table 2:** Solvents used in this study.

No.	Solvent Composition		Molar Ratio	Water Content	Viscosity (mPa·s)	Density (g·mL^−1^)	Extraction Efficiency (mg·g^−1^)
	HBAs	HBDs					
ChCl−Ur	Choline chloride	Urea	1:1	20%	49.80 ± 0.26 ^c^	1.21	41.38 ± 1.12 ^f^
ChCl−Eg		Ethylene glycol	1:1	20%	18.03 ± 0.17 ^j^	1.17	59.22 ± 0.57 ^c^
ChCl−Pr		1,3-Propanediol	1:1	20%	24.71 ± 0.19 ^h^	1.42	62.47 ± 0.52 ^b^
ChCl−Gl		Glycerol	1:1	20%	44.54 ± 0.12 ^d^	1.20	53.39 ± 0.40 ^d^
ChCl−Bu		1,4-Butanediol	1:1	20%	30.60 ± 0.11 ^f^	1.12	53.52 ± 1.44 ^d^
ChCl−Ao		Oxalic acid	1:1	20%	18.61 ± 0.10 ^i^	1.23	53.24 ± 0.98 ^d^
ChCl−La		Lactic acid	1:1	20%	29.01 ± 0.11 ^g^	1.18	66.31 ± 2.64 ^a^
ChCl−Ma1		Malonic acid	1:1	20%	35.95 ± 0.10 ^e^	1.18	62.17 ± 2.26 ^b^
ChCl−Ma2		Malic acid	1:1	20%	195.13 ± 0.24 ^b^	1.28	61.16 ± 1.12 ^bc^
ChCl−Ca		Citric acid	1:1	20%	218.44 ± 0.23 ^a^	1.30	46.52 ± 1.63 ^e^
60% ET	-						54.61 ± 0.68 ^d^

Note: viscosity and extraction efficiency measurements were repeated three times and are expressed as mean ± standard error; different letters indicate significant differences (*p* < 0.05).

## Data Availability

The data presented in this study are available on request from the corresponding authors. The data are not publicly available due to privacy restrictions.

## Supplementary Materials

The following supporting information can be downloaded at: https://www.mdpi.com/article/10.3390/foods14040558/s1. Figure S1: The calibration curve of standard rutin. Figure S2: Figures a–r show the FT-IR and ^13^C NMR plots of the solvents used in this study, respectively. Figure S3: ^13^C NMR maps of ChCl−La groups with different molar ratios. Figure S4: Cyclic experiment with DES. Table S1: Independent variables and levels for Box–Behnken design. Table S2: Box–Behnken design and experimental results of HCFs. Table S3: ANOVA results of the models for HCF flavonoid extraction yields of HCFs. Table S4: Comparison of flavonoid extraction [36,48,55,56,57].
