# Supplementary material for: Deep Eutectic Solvent-Based Ultrasound-Assisted Extraction of Flavonoids from Houttuynia cordata
Source: Foods. 2025 Feb 7;14(4):558. doi: 10.3390/foods14040558 (PMC11854880; doi:10.3390/foods14040558)
Supplement: Supplementary file 1 [file foods-14-00558-s001.zip › foods-3409058-supplementary.pdf]

Supplementary Materials

Deep Eutectic Solvent-Based Ultrasound-Assisted Extraction of Flavonoids from *Houttuynia cordata*

Xinxin Wu [1], Ling Yan [2,3], Jingda Li [1,*] and Zhijian Tan [2,*]

[1] College of Life Sciences, Yangtze University, Jingzhou 434025, China; wuxinxin20242024@163.com
[2] Institute of Bast Fiber Crops & Center of Southern Economic Crops, Chinese Academy of Agricultural Sciences, Changsha 410205, China; yanling7711@126.com
[3] College of Life and Environmental Science, Hunan University of Arts and Science, Changde 415000, China
* Correspondence: jingdali_shenqi@163.com (J.L.); tanzhijian@caas.cn (Z.T.)

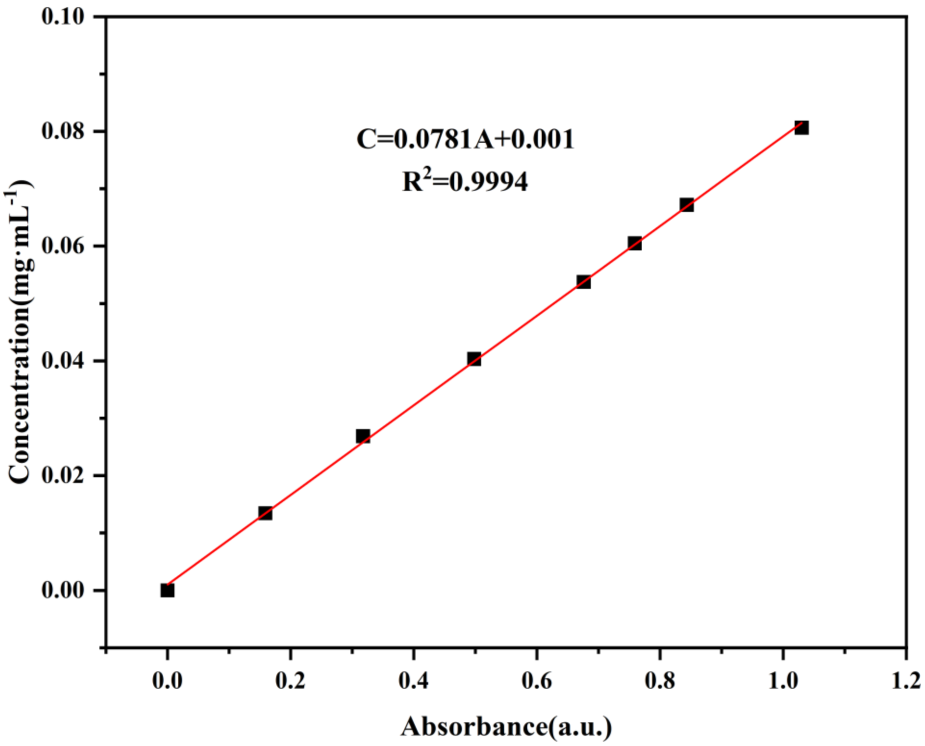

Figure S1 The calibration curve of standard rutin

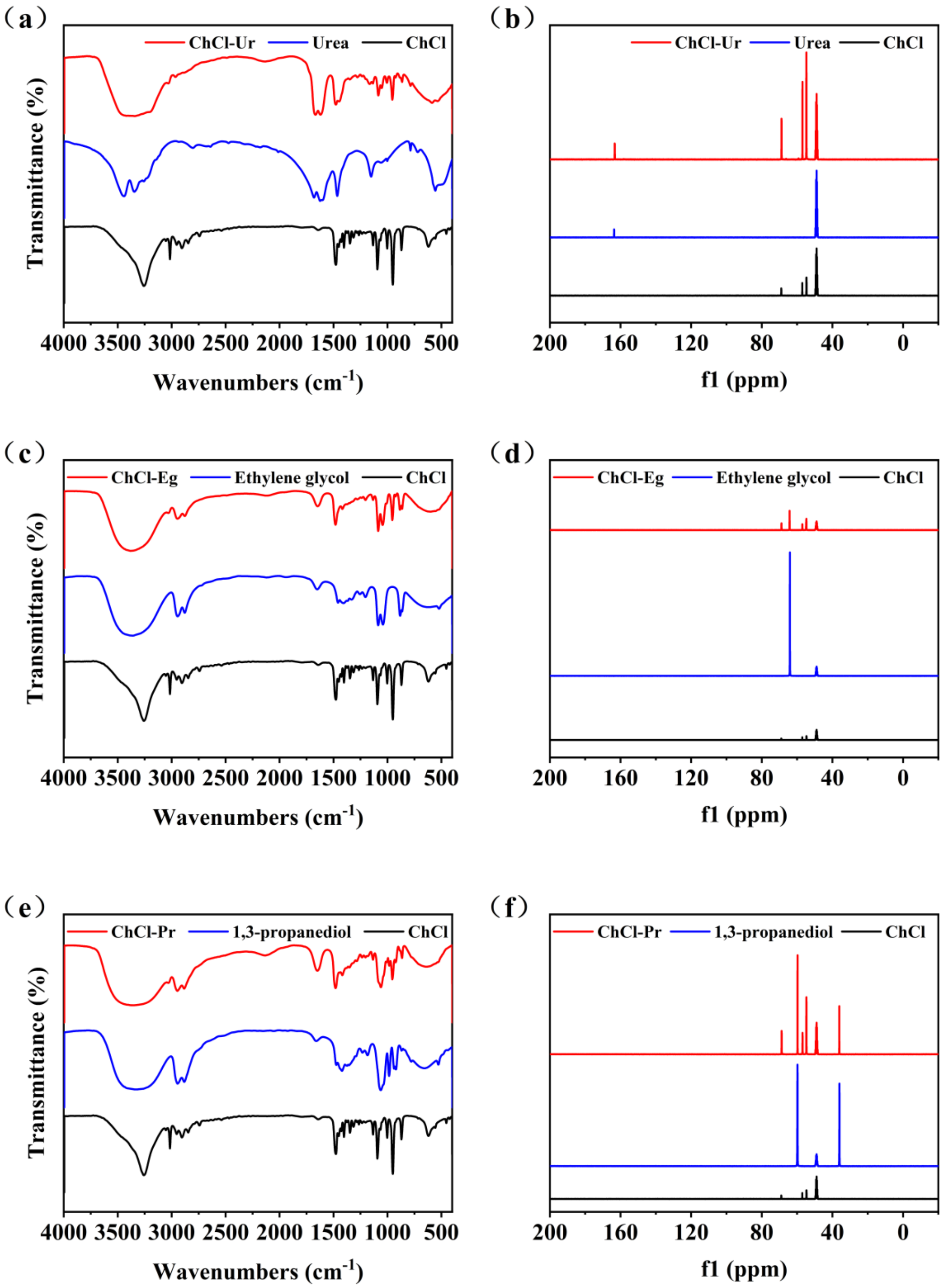
（a）
ChCl-Ur
Urea
ChCl
Transmittance (%)
4000 3500 3000 2500 2000 1500 1000 500
Wavenumbers (cm-1)
（b）
ChCl-Ur
Urea
ChCl
200 160 120 80 40 0
f1 (ppm)
（c）
ChCl-Eg
Ethylene glycol
ChCl
Transmittance (%)
4000 3500 3000 2500 2000 1500 1000 500
Wavenumbers (cm-1)
（d）
ChCl-Eg
Ethylene glycol
ChCl
200 160 120 80 40 0
f1 (ppm)
（e）
ChCl-Pr
1,3-propanediol
ChCl
Transmittance (%)
4000 3500 3000 2500 2000 1500 1000 500
Wavenumbers (cm-1)
（f）
ChCl-Pr
1,3-propanediol
ChCl
200 160 120 80 40 0
f1 (ppm)

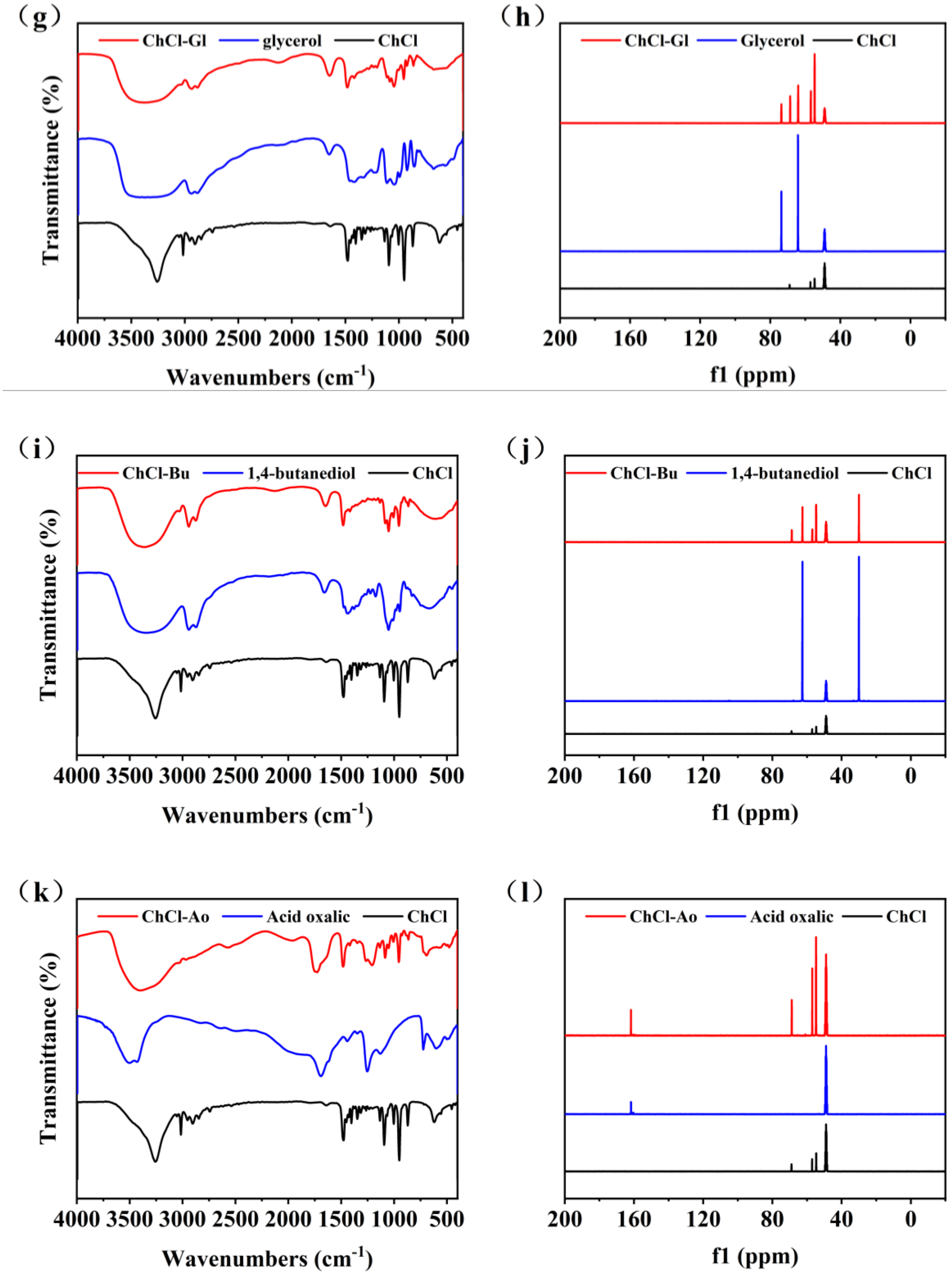

(g)
ChCl-Gl
glycerol
ChCl
Transmittance (%)
4000 3500 3000 2500 2000 1500 1000 500
Wavenumbers (cm-1)
(h)
ChCl-Gl
Glycerol
ChCl
200 160 120 80 40 0
f1 (ppm)
(i)
ChCl-Bu
1,4-butanediol
ChCl
Transmittance (%)
4000 3500 3000 2500 2000 1500 1000 500
Wavenumbers (cm-1)
(j)
ChCl-Bu
1,4-butanediol
ChCl
200 160 120 80 40 0
f1 (ppm)
(k)
ChCl-Ao
Acid oxalic
ChCl
Transmittance (%)
4000 3500 3000 2500 2000 1500 1000 500
Wavenumbers (cm-1)
(l)
ChCl-Ao
Acid oxalic
ChCl
200 160 120 80 40 0
f1 (ppm)

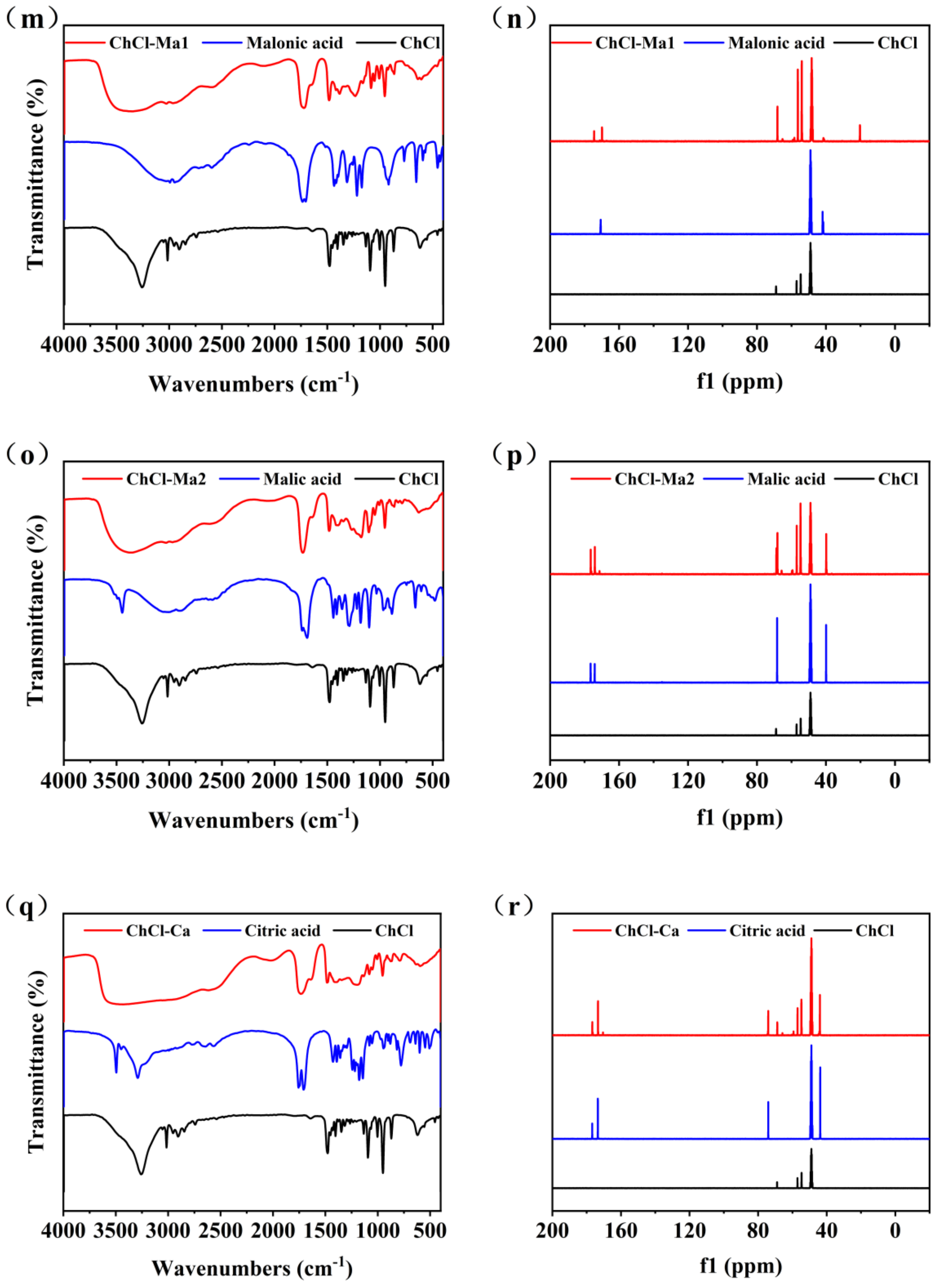

Figure S2 Figures a-r show the FT-IR and ^{13}C NMR plots of solvents used in this study, respectively.

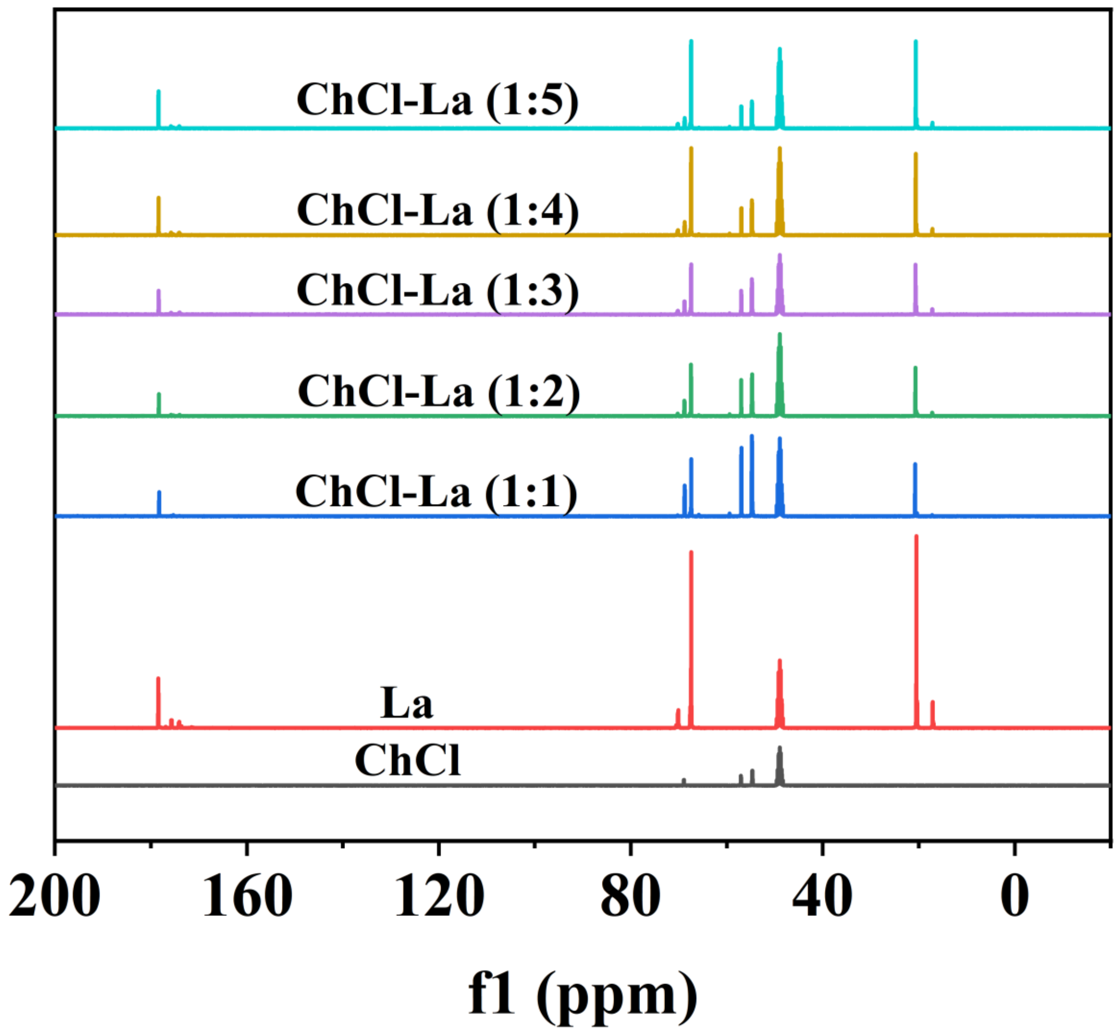

Figure S3 ^{13}C NMR maps of ChCl-La groups with different molar ratios.

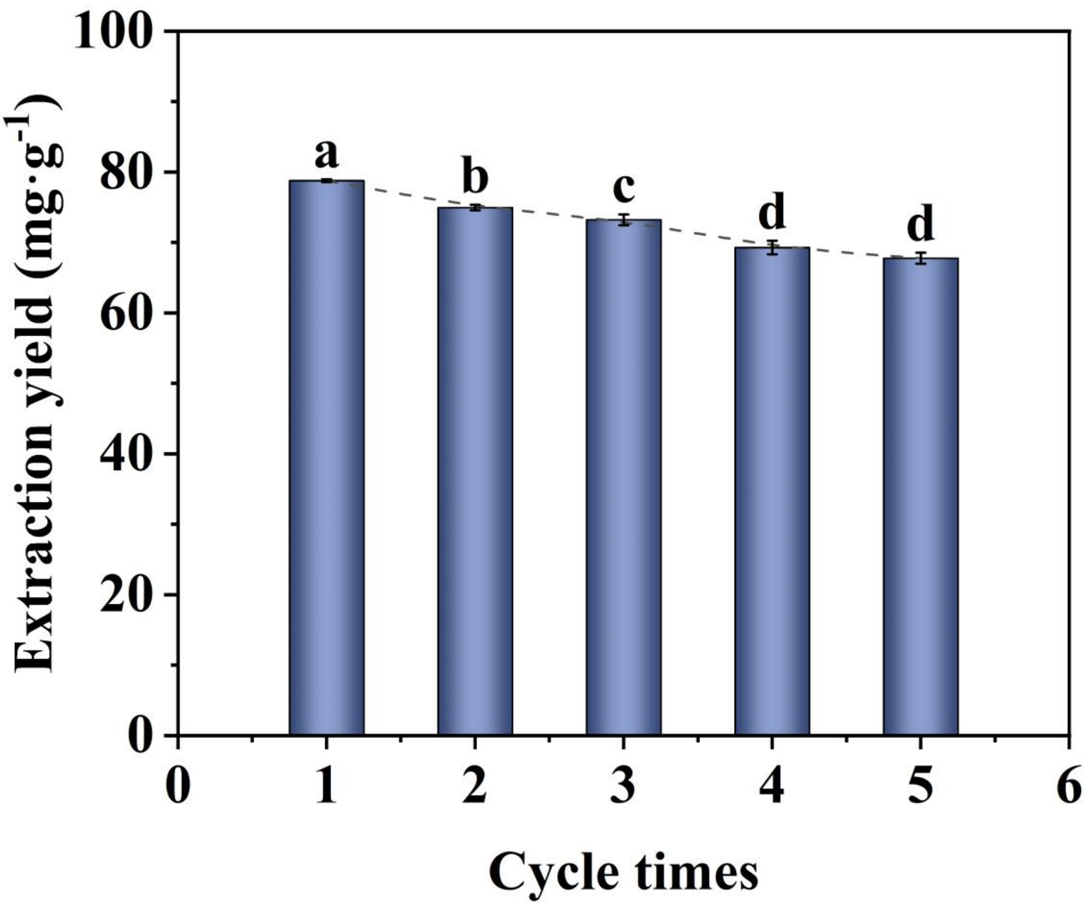

Figure S4 Cyclic experiment with DES.

Table S1 Independent Variables and Levels for Box-Behnken Design.

No.	Variables	Unit	Level		
			-1	0	1
A	solid-liquid ratio	$g·mL^{-1}$	1:10	1:20	1:30
B	molar ratio	HBA: HBD	1:1	1:2	1:3
C	ultrasonic power	W	350	420	490

Table S2 Box-Behnken design and experimental results of HCF.

Run	A	B	C	Extraction yield%
	mg/mL	HBA/HBD	W	
1	20	1:2	420	81.75
2	10	1:3	420	62.36
3	20	1:2	420	80.86
4	20	1:1	490	71.02
5	10	1:2	350	65.39
6	30	1:1	420	68.10
7	10	1:2	490	66.56
8	10	1:1	420	63.70
9	20	1:1	350	69.17
10	20	1:3	490	71.29
11	20	1:2	420	81.62
12	20	1:2	420	81.22
13	20	1:3	350	70.15
14	20	1:2	420	80.68
15	30	1:2	490	70.45
16	30	1:3	420	69.60
17	30	1:2	350	69.32

Table S3 ANOVA results of the models for HCF flavonoid extraction yields of HCF.

Source	Sum of squares	freedom	Mean square	F value	P value Prob > F	
Model	702.23	9	78.03	188.49	< 0.0001	Significant
A	47.37	1	47.37	114.44	< 0.0001	
B	0.2475	1	0.2475	0.5979	0.4647	
C	3.52	1	3.52	8.50	0.0225	
AB	2.02	1	2.02	4.87	0.0630	
AC	0.0003	1	0.0003	0.0008	0.9784	
BC	0.1245	1	0.1245	0.3007	0.6005	
A^2	332.12	1	332.12	802.30	< 0.0001	
B^2	172.72	1	172.72	417.23	< 0.0001	
C^2	82.12	1	82.12	198.38	< 0.0001	
Residual	2.90	7	0.4140			
Lack of fit	2.04	3	0.6785	3.15	0.1485	Not significant
Pure error	0.8623	4	0.2156			
Sum	705.13	16				
R^2	0.9959					
R_{Adj}^2	0.9906					
C.V.%	0.8942					

Table S4 Comparison of flavonoid extraction

**No.**	Extraction solvent	**Type**	**Extraction efficiency ($mg \cdot g^{-1}$)**	**Source**
1	ChCl-La	Houttuynia cordata flavonoids	79.86	This study
2	Betaine-La	Sea buckthorn fruits' flavonoids	5.248	[1]
3	ChCl-Citric acid	Potentilla fruticosa L. flavonoids	50.26	[2]
4	Ethylene glycol-Glycolic acid	Mung Bean flavonoids	2.34	[3]
5	1, 4-butanediol-Acetic acid	Xanthoceras sorbifolia Bunge flavonoids	30.37	[4]
6	Tetrapropylaminium bromide-1,4-butanediol	Selaginella chaetoloma total biflavonoids	21.68	[5]

References

1. Ma, P.; Li, Z.; Jin, Y.; Zuo, J.; Zhang, Y.; Dong, A.; Xiao, D.; Burenjargal, M. Green and Efficient Extraction Process of Flavonoids from Sea Buckthorn Fruits by Natural Deep Eutectic Solvents Aided with Ultrasound. *Microchemical Journal* **2024**, *205*, 111265, doi:10.1016/j.microc.2024.111265.
2. Xue, H.; Li, J.; Wang, G.; Zuo, W.; Zeng, Y.; Liu, L. Ultrasound-Assisted Extraction of Flavonoids from Potentilla Fruticosa L. Using Natural Deep Eutectic Solvents. *Molecules* **2022**, *27*, 5794, doi:10.3390/molecules27185794.
3. Gao, J.; Xie, L.; Peng, Y.; Li, M.; Li, J.; Ni, Y.; Wen, X. Deep Eutectic Solvents as New Extraction Media for Flavonoids in Mung Bean. *Foods* **2024**, *13*, 777, doi:10.3390/foods13050777.

4. Feng, Z.; Yang, D.; Guo, J.; Bo, Y.; Zhao, L.; An, M. Optimization of Natural Deep Eutectic Solvents Extraction of Flavonoids from *Xanthoceras Sorbifolia Bunge* by Response Surface Methodology. *Sustainable Chemistry and Pharmacy* **2023**, *31*, 100904, doi:10.1016/j.scp.2022.100904.
5. Liu, C.; Qiao, L.; Gao, Q.; Zhang, F.; Zhang, X.; Lei, J.; Ren, M.; Xiao, S.; Kuang, J.; Deng, S.; et al. Total Biflavonoids Extraction from Selaginella Chaetoloma Utilizing Ultrasound-Assisted Deep Eutectic Solvent: Optimization of Conditions, Extraction Mechanism, and Biological Activity in Vitro. *Ultrasonics Sonochemistry* **2023**, *98*, 106491, doi:10.1016/j.ultsonch.2023.106491.
